# Long-term safety and efficacy of deoxycytidine/deoxythymidine in treatment of POLG-related disorders

**DOI:** 10.1016/j.neurot.2026.e00945

**Published:** 2026-06-15

**Authors:** Anthony C.T. Cheung, Saoussen Berrahmoune, Christelle Dassi, Heather Pekeles, Tommy Gagnon, Paula J. Waters, Ralf Eberhard, Daniela Buhas, Kenneth A. Myers

**Affiliations:** a Division of Medical Genetics, Department of Specialized Medicine, McGill University Health Centre, 1001 Décarie Boulevard, Montreal, Quebec, H4A 3J1, Canada; b Child Health and Human Development Program, Research Institute of the McGill University Health Centre, 2155 Guy Street, Suite 500, Montreal, Quebec, H3H 2R9, Canada; c Division of Neurology, Department of Pediatrics, Montreal Children's Hospital, McGill University Health Centre, 1001 Décarie Boulevard, Montreal, Quebec, H4A 3J1, Canada; d Medical Genetics Service, Department of Laboratory Medicine, CHUS and Department of Pediatrics, Université de Sherbrooke, Sherbrooke, Quebec, J1K 2R1, Canada; e Centre de Recherche du Centre Hospitalier Universitaire de Sherbrooke (CRCHUS), 12e Avenue N Porte 6, Sherbrooke, Quebec, J1H 5N4, Canada; f Department of Human Genetics, McGill University, Montreal, Quebec, Canada; g Department of Neurology and Neurosurgery, Montreal Children's Hospital, McGill University Health Centre, 1001 Décarie Boulevard, Montreal, Quebec, H4A 3J1, Canada

**Keywords:** DNA polymerase gamma, POLG, Deoxynucleoside, Mitochondrial disorder, Mitochondrial DNA depletion disorder

## Abstract

We evaluated the safety and efficacy of enteral deoxycytidine/deoxythymidine combination therapy in treatment of POLG-related disorders, genetic mitochondrial diseases characterized by progressive neurological degeneration. A single-centre open-label phase II trial was conducted. Inclusion criteria included: age 3 months to 60 years, clinical diagnosis of POLG-related disorder, and biallelic pathogenic *POLG* variants. Participants received deoxycytidine/deoxythymidine initially at 100 mg/kg/day (50 mg/kg deoxycytidine and 50 mg/kg deoxythymidine), titrated to 400 mg/kg/day over three weeks. The current protocol is a 60-month treatment period with primary outcomes the Newcastle Mitochondrial Disease Scale sections I-III and serum growth differentiation factor 15. Secondary outcomes include quality of life questionnaires, seizure diary, EEG, and blood and urine laboratory tests assessing end organ function. Outcomes were assessed at baseline, 1-month, 2-month, 3-month, and 6-month timepoints, then every 6 months thereafter. Twenty-five individuals (14 male, 11 female; mean age 12.3 years) started deoxycytidine/deoxythymidine. Five died during the trial and five withdrew. The most common treatment-related adverse event was diarrhea. Newcastle Mitochondrial Disease Scale sections I-III score decreased (improved) from baseline at all timepoints from 1 month to 24 months (p < 0.05). Serum growth differentiation factor 15 significantly decreased (improved) from baseline at 1-month, 2-month, and 3-month timepoints (p < 0.05). Quality of life score improved at 3-month, 12-month, and 18-month timepoints (p < 0.05). In summary, our data suggest deoxycytidine/deoxythymidine is safe and effective for POLG-related disorders; however, further study is needed to clarify the therapeutic mechanism(s) so that the treatment can be refined and optimized.

## Introduction

*POLG* (OMIM 174763) encodes the catalytic subunit of DNA polymerase gamma, an enzyme critical for mitochondrial DNA replication and repair [1,2]. Pathogenic variants in *POLG* are associated with a group of conditions with overlapping phenotypes including seizures, developmental impairment and regression, ataxia, neuropathy, myopathy, and hepatic dysfunction [1,3]. POLG-related disorders are sometimes divided into distinct syndromes – Alpers-Huttenlocher syndrome, childhood myocerebrohepatopathy spectrum, myoclonic epilepsy myopathy sensory ataxia, ataxia neuropathy spectrum, and autosomal recessive and autosomal dominant progressive external ophthalmoplegia – however, there is often overlap between these syndromes, and some individuals with biallelic *POLG* pathogenic variants do not clearly fit it into any one [1,3].

Although there are currently no approved treatments for POLG-related disorders, treatment with deoxynucleosides has been proposed for other mitochondrial DNA depletion disorders. Deoxycytidine (dC) and deoxythymidine (dT) therapy has been shown to improve clinical outcomes in thymidine kinase 2 (TK2) deficiency [[4], [5], [6], [7]], and recently received FDA approval. As part of a broader phase 2 open label study, we previously reported preliminary data on the safety and efficacy of dC/dT in 10 individuals with pediatric-onset POLG-related disorders [8]. This report provides data on the long-term safety and efficacy of pyrimidine deoxynucleosides from a larger POLG cohort that includes patients with adult-onset disease.

## Materials and Methods

### Study design and participants

This is a single-centre open-label phase II single arm trial to assess safety, tolerability, and efficacy of a 50:50 mixture of dC and dT, administered enterally, for people with POLG-related disorders. The trial is registered with ClinicalTrials.gov, NCT04802707 (https://clinicaltrials.gov/ct2/show/NCT04802707). The study is based at the McGill University Health Centre, Glen Site. A data safety monitoring board reviews data at approximately 6-month intervals. All data are securely stored on REDCap, hosted by the Research Institute of the McGill University Health Centre. Inclusion criteria for the study are age 3 months to 60 years at time of enrollment (∗ the upper age limit was initially 18 years, later increased to 60 years in a protocol amendment), clinical diagnosis of a POLG-related disorder, and having biallelic pathogenic or likely pathogenic variants in *POLG*. This is a sub-study of a broader trial exploring the safety and efficacy of dC/dT for mitochondrial DNA depletion disorders of various genetic causes, including pathogenic variants in any of *POLG*, *RRM2B*, *C10Orf2*, *MPV17*, *SUCLA2*, *SUCLG1*, or *FBXL4*. Exclusion criteria are known hypersensitivity to dC or dT, inability to receive the product enterally (i.e., orally or via nasogastric or gastrostomy tube), and inability to provide informed consent (from patient or legal representative). Participant race/ethnicity was collected due to the genetic nature of the disorder being studied and was determined by self-report from patient or caregivers. Here, we present an interim analysis of data collected from October 14, 2021 to October 15, 2025.

### Procedures

All participants receive the same drug intervention. dC and dT are obtained from Biosynth (United Kingdom) or Alfa Chemistry (United States) in powder form, dissolved in water and distributed in three enterral portions over the day. Dosing is 100 mg/kg/day (50 mg/kg dC and 50 mg/kg dT) in week #1, 200 mg/kg/day in week #2, 300 mg/kg/day in week #3, and then 400 mg/kg/day for the remaining period. This dose is at the low end of what has been used previously in studies for TK2 deficiency [5,6], and was chosen due to the uncertain safety of the intervention in this patient population. If the maximum dose of 400 mg/kg/day is not tolerated, then the dose is reduced until tolerated. The initial protocol was for a 6-month treatment period, subsequently amended to 24 months, and later 60 months, after some participants had significant positive responses and wished to continue the therapy but had no other avenue to do so.

The Newcastle Mitochondrial Disease Scale (NMDS) is a validated clinical scale used to assess clinical status in people with mitochondrial disease [[9], [10], [11]]. There are four versions based on age: 0–24 months, 2–11 years, 12–18 years, and >18 years. There are four sections to the NMDS: Section I – current function, Section II – system specific involvement, Section III – current clinical assessment, and Section IV – quality of life. This latter section is a questionnaire completed by the patient or caregivers. The 0–24 months, 2–11 years, and 12–18 years versions of the NMDS have a Quality of Life questionnaire included, while the adult version only recommends using a separate scale, the Short-Form 12-Item Version 2 [12]. For patients aged 18 years or younger, we assessed quality of life with the NMDS section IV, while for patients >18 years, we used the Wisconsin Quality of Life Client Questionnaire (WQLCQ), a validated assessment tool that includes multiple dimensions of life quality [13]. As recommended in the test manual, NMDS Section IV was analyzed separately from the disease score obtained in sections I-III [10,11]. Patients are scored on the NMDS at baseline, 1-month, 3-month, 6-month, and every subsequent 6-month visit by a clinical geneticist. In the event that the patient is unable to travel to the McGill University Health Centre for an in-person assessment, the scoring of the NMDS is done virtually via video teleconference.

Growth differentiation factor 15 (GDF-15) is a blood serum test validated as a quantitative biomarker of mitochondrial dysfunction. It has been used as a biomarker of treatment response in many studies of mitochondrial disease, including some involving TK2 deficiency [14]. Serum GDF-15 levels are collected at baseline and at every subsequent trial visit, with analysis performed at Mayo Clinic Laboratories (Rochester, MN).

Blood and urine samples are collected at baseline, and all subsequent visits, to monitor for any concerning deteriorations in end organ function (i.e., liver, kidney, muscle) and overall mitochondrial function. Biochemical evaluations include complete blood count (CBC), serum aspartate aminotransferase (AST), alanine aminotransferase (ALT), gamma-glutamyl transferase (GGT), bilirubin, albumin, creatinine, urea, electrolytes, creatine kinase (CK), capillary/venous blood gas, lactate, plasma amino acids, plasma acylcarnitine profile, urine amino acids, urine purines and pyrimidines. Routine EEG studies are performed to assess cerebral activity at baseline, 3 months, 6 months, and every subsequent 6-month interval; a qualified epileptologist (KAM) evaluates whether there has been a significant change in frequency of epileptiform discharges or background activity abnormalities (slowing and organization). All patient-reported adverse events are recorded.

### Outcomes

In the current protocol, the two primary outcome measures are NMDS score (sections I-III) and serum GDF-15 level. Secondary outcomes include biochemical tests, quality of life (NMDS section IV for pediatric patients and WQLCQ for adult patients), seizure diary, EEG, and patient-reported adverse events.

### Statistical analysis

As this trial was initially primarily aimed at determining the safety of dC/dT for POLG-related disorders, we did not perform a formal sample size calculation. Our initial recruitment goal was five patients, given the rarity of POLG-related disorders. A one-tailed Wilcoxon signed rank test was used to assess changes in NMDS scores and GDF-15 values from baseline to each timepoint, as we hypothesized dC/dT would elicit clinical benefit. A two-tailed Wilcoxon signed rank test was used to assess for changes in all other biochemical tests as these were primarily followed for safety reasons; we did not know if there would be baseline abnormalities, and there was no hypothesis as to what change, if any, would be expected. p values given are descriptive, given the exploratory nature of this study. To evaluate quality of life from the overall cohort, we combined the pediatric NMDS section IV data and adult WQLCQ data into a normalized quality of life score, expressed on a scale from 0 to 10 (details in supplementary material). In a post hoc secondary analysis, we also examined whether patterns of response were related to age of symptom onset, individually analyzing data for patients with onset at less than 12 years of age, 12–30 years, or after age 30 years.

*Ethics*: The study was approved by the McGill University Health Centre Research Ethics Board (2021–7654), with a no objection letter from Health Canada. Informed consent was obtained from participants or parents prior to enrollment.

## Results

**Fig. 1:**
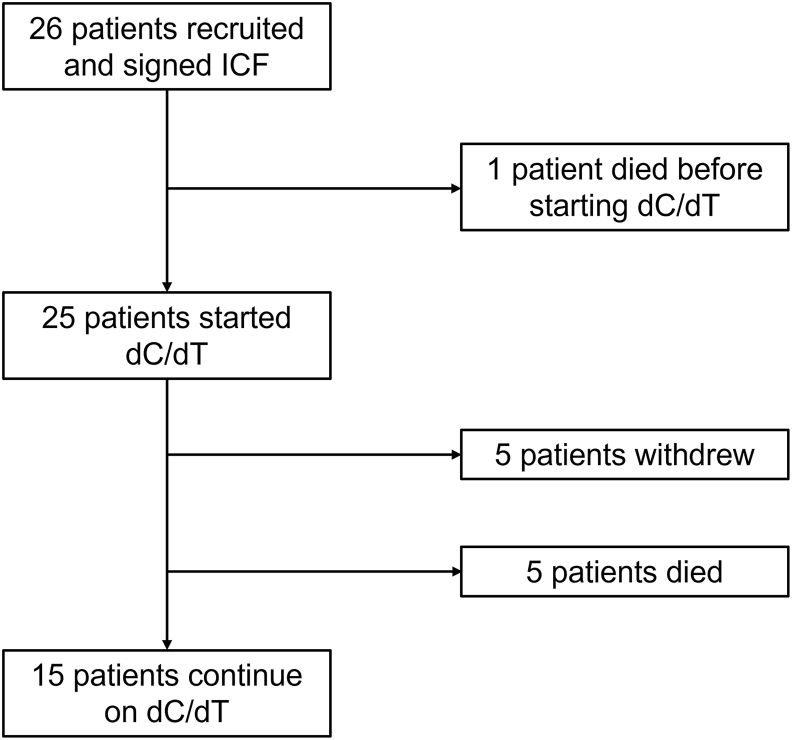
Flow chart of participants.

Twenty-five patients (14 males, 11 females) with POLG-related disorders meeting inclusion/exclusion criteria were enrolled and started dC/dT (Table 1, individual patient details in supplementary material). Nineteen (76%) had symptom onset before 12 years of age, four (16%) between age 12 and 30 years, and two (8%) after age 30 years. Recruitment and enrollment are summarized in a flow chart (Fig. 1). Mean age at dC/dT initiation was 12.3 y (standard deviation (SD) 12.5 y, range 1.0–47.9 y). One additional patient was screened and consented to enrollment but died before being able to travel to Montreal for the baseline visit. Duration of dC/dT treatment at last follow-up ranged from 2 to 46 months, with a total of 530 months of cumulative exposure.

**Table 1:** Baseline demographics and clinical features.

Variable	
Age at enrollment	Mean 12.3 y (SD 12.5 y; range 1.0–47.9 y)
Sex	14 M, 11 F
Duration of dC/dT treatment at last follow-up	Mean 21.2 mths (SD 14.1 mths; range 2–46 mths)
Signs/Symptoms	
- *Epilepsy*	17
*- Ataxia*	10
*- Ptosis/ophthalmoplegia*	8
- *Liver dysfunction*	3
Age of symptom onset	
- *< 12 y*	19
*12–30 y*	4
*- >30 y*	2

Five patients (20%) withdrew from the trial. Patient #5 was a 3-year-old girl at the time of enrollment; at that point, she had only photophobia and constipation, having received a genetic diagnosis because of her more severely affected older brother. The family elected to withdraw just before the 12-month visit due to the challenges of traveling to and from the study site. Patient #11 was a 12-month-old boy at the time of enrollment, with Alpers-Huttenlocher phenotype. The family withdrew just before the 12-month visit due to the challenges of travel to and from the study site, noting concerns that the patient might become ill during the trip. Patient #6 was a 17-year-old female at the time of enrollment, with ataxia-neuropathy spectrum. She withdrew just before the 30-month visit as the family decided it would be more convenient and financially viable to order dC/dT directly from a supplier and continue the treatment under the supervision of their primary medical team. Patient #19 was a 21-year-old male with epilepsy and developmental regression; he also had a diagnosis of ulcerative colitis, and his family became concerned that the dC/dT might exacerbate the condition, so withdrew after 2 months. Patient #15 was a boy with mild speech and motor delay; his family withdrew because they were no longer able to cover the costs of travel and accommodation for trial visits.

### Newcastle Mitochondrial Disease Scale sections I-III

**Fig. 2:**
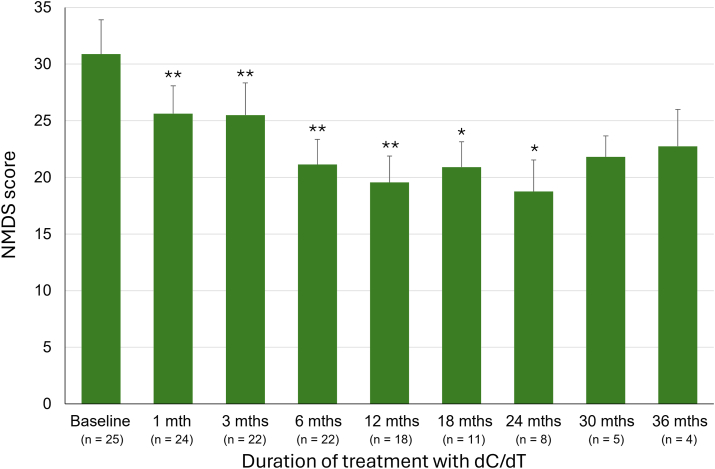
Newcastle Mitochondrial Disease Scale parts I-III scores. Statistically significant reductions in NMDS I-III from baseline were seen at the 1-month, 3-month, 6-month, 12-month, 18-month, and 24-month timepoints. Note that because this is an ongoing trial, the number of data points is different for each timepoint (e.g., the 1-month timepoint is based on data from 24 patients, while 36-month timepoint comes from only 4. Error bars reflect standard error. ∗∗ = p < 0.001, ∗ = p < 0.05.

The mean NMDS I-III scores at baseline and subsequent timepoints are shown (Fig. 2 and Table 2) with patient-specific details in supplementary material. NMDS sections I-III scores decreased from 30.9 at baseline to 25.6 at one month (estimated difference 3.5; 95% confidence interval (CI) 2.0 to ∞; p < 0.001), 25.5 at 3 months (estimated difference 5.5; 95% CI 3.0 to ∞; p < 0.001), 21.1 at 6 months (estimated difference 6.5; CI 4.0 to ∞; p < 0.001), 19.6 at 12 months (estimated difference 7.5; 95% CI 5.5 to ∞; p < 0.001), 20.9 at 18 months (estimated difference 10.0; 95% CI 6.0 to ∞; p = 0.003), and 18.8 at 24 months (estimated difference 10.9; 95% CI 6.0 to ∞; p = 0.011).

**Table 2:** Key Outcome Measures Up To 24 Months of dC/dT Treatment. Values are shown±standard deviation. Values are bolded if p < 0.05 for comparison to baseline. ∗ Data not shown as only 1 or 0 values for WQLCQ. Abbreviations: GDF-15 = growth differentiation factor 15; ND = no data collected at this timepoint; NMDS = Newcastle mitochondrial disease scale; QOL = quality of life; WQLCQ = Wisconsin Quality of Life Client Questionnaire.

Variable	Baseline	1 month	2 months	3 months	6 months	12 months	18 months	24 months
NMDS section 1-III	30.9 ± 14.9	25.6 ± 12.3 (p < 0.001)	ND	25.5 ± 13.3 (p < 0.001)	21.1 ± 10.4 (p < 0.001)	19.6 ± 9.9 (p < 0.001)	20.9 ± 7.4 (p = 0.003)	18.8 ± 7.9 (p = 0.011)
GDF-15 (pg/ml)	1164.5 ± 1031	856 ± 430 (p = 0.049)	789 ± 548 (p = 0.004)	1197 ± 1383 (p = 0.03)	949 ± 708 (p = 0.17)	1321 ± 1622 (p = 0.055)	1301 ± 1221 (p = 0.34)	945 ± 931 (p = 0.22)
NMDS section IV (pediatric patients only)	13.6 ± 4.4	13.7 ± 3.5 (p = 0.28)	ND	12.9 ± 3.9 (p = 0.14)	12.8 ± 3.6 (p = 0.26)	11.7 ± 5.4 (p = 0.15)	12.4 ± 5.6 (p = 0.065)	11.0 ± 3.3 (p = 0.055)
WQLCQ (adult patients only)	1.02 ± 0.77	1.35 ± 0.77 (p = 0.031)	ND	1.54 ± 0.63 (p = 0.063)	1.49 ± 0.62 (p = 0.063)	1.38 ± 0.37 (p = 0.125)	∗	∗
Normalized QOL (combined NMDS section IV and WQLCQ)	3.96 ± 2.14	3.67 ± 1.71 (p = 0.070)	ND	3.39 ± 1.47 (p = 0.021)	3.34 ± 1.50 (p = 0.059)	3.30 ± 1.64 (p = 0.041)	3.07 ± 1.47 (p = 0.042)	∗

### Growth differentiation factor 15

Serum GDF-15 levels were significantly lower at the 1-month, 2-month, and 3-month timepoints compared to baseline (Table 2, supplementary material). Non-significant trends of decrease were observed at all other timepoints. Unfortunately, data were not available for all patients at all timepoints; some samples could not be processed due to specimen handling issues and some in-person patient visits had to be canceled because patients were unable to travel. An additional challenge was that the commercial laboratory that processes the samples has an upper reporting limit of 6000 pg/ml, and some patients had values consistently above that limit.

### Quality of Life

**Fig. 3:**
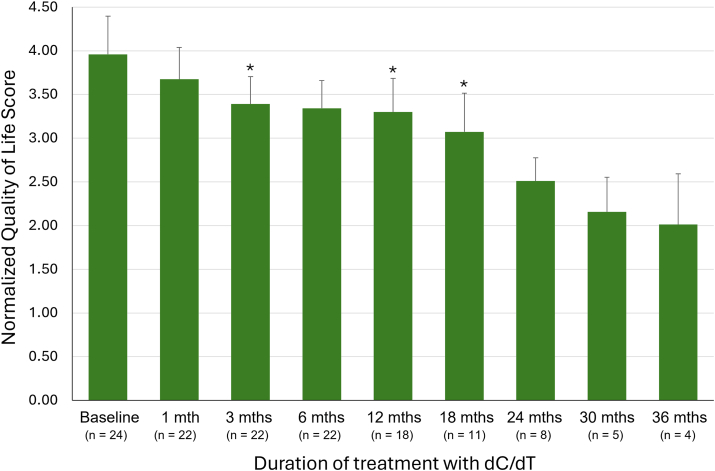
Normalized Quality of Life Score. Statistically significant reductions (improvements) in normalized quality of life score from baseline were seen at the 3-month, 12-month, and 18-month timepoints. Note that because this is an ongoing trial, the number of data points is different for each timepoint (e.g., the 1-month timepoint is based on data from 22 patients, while 36-month timepoint comes from only 4). Error bars reflect standard error. ∗ = p < 0.05.

There were no significant differences in mean NMDS section IV scores for pediatric patients (Table 2, supplementary material); however, trends of improvement were seen at all timepoints, often very nearly meeting the threshold of significance (e.g. p values of 0.055–0.065 at 18-month, 24-month, 30-month, and 36-month timepoints). For patients >18 years, WQLCQ scores showed a significant increase (improvement) at 1 month and approached significance at all other timepoints (Table 2, supplementary material). When quality of life data for pediatric and adult patients were combined in the normalized quality of life score, improvements from baseline were significant at the 3-month, 12-month, and 18-month timepoints (Fig. 3, Table 2, supplementary material).

### Seizures

The details of seizure patterns are in supplementary material. 17/25 patients (68%) had a history of seizures prior to enrolling in the trial. Of the eight with no prior seizure history, none developed seizures after starting the trial. Of the patients with a history of epilepsy, 3/17 (18%) were considered seizure-free at the time of enrollment (no seizures in previous 6 months); those individuals all remained seizure-free for the remainder of their time in the trial. Of the patients with uncontrolled seizures at the time of enrollment, 10/14 (71%) had >50% improvement in seizures, including three who were seizure-free at last review. No patients had clear worsening of seizures.

### Serious adverse events

Five patients (20%) died during the trial. Patient #10 was a 2-year-old male at the time of enrollment, with seizures and developmental regression (Alpers-Huttenlocher phenotype). Ten months after dC/dT initiation, he was hospitalized for increased seizures and vomiting and was treated for a presumed aspiration pneumonia. During his admission, he developed multi-organ dysfunction, hypothermia, progressive feeding intolerance, and severe acute kidney injury/anuria, and subsequently died.

Patient #12 was a 6-year-old boy at the time of enrollment, with ataxia-neuropathy spectrum and associated ptosis, weakness, motor regression, and areflexia; he died 15 months after starting the trial. The autopsy identified an influenza B infection with acute bronchopneumonia as the cause of death.

Patient #18 was a 2-year-old female who had severe liver failure and drug-resistant epilepsy at the time of enrollment. The patient had a planned admission to the pediatric ICU post-trial initiation for further management of her liver dysfunction. At 1-month post-dC/dT initiation, she developed bilateral subdural hematomas in the context of coagulopathy that required surgical evacuation and multiple blood products. She also developed sepsis from *Klebsiella pneumoniae* bacteremia requiring intubation for 10 days. At 2.5 months post-dC/dT initiation, she had increased seizures and worsening respiratory distress from a presumed infection and required intubation. Due to worsening liver dysfunction and mental status, the family requested hospice care. The patient died 3 months after enrollment, several days after discharge and extubation.

Patient #25 was a 39-year-old female at the time of enrollment, with ptosis, chronic progressive external ophthalmoplegia, peripheral neuropathy, ataxia, and dystonia. She died in her sleep after 3 months in the trial, with no clear cause identified.

Patient #7 was an 11-year-old female at the time of enrollment, with Alpers-Huttenlocher syndrome and severe spastic quadriplegia. She had been inadvertently treated with valproic acid early in her clinical course (prior to starting the trial), which likely contributed to her severe disability. She died in her sleep after 37 months in the trial. She was apparently well at the time of death; an autopsy was not performed, so the death was considered probable sudden unexpected death in epilepsy (SUDEP).

Patients 2, 9, and 11 had 1, 3, and 1 hospitalization(s), respectively, for deterioration in the context of intercurrent viral illnesses. Patient 11 also had an elective admission for a G-tube insertion. Patient 3, who had a previous liver transplant, required admission to hospital on one occasion with increased serum liver enzymes and fever not thought to be related to dC/dT treatment. She was ultimately diagnosed with bile duct and small intestine obstruction which required over 6 surgeries including surgical drain placement. Patient 5 who had intermittent constipation prior to starting the trial, was admitted to hospital for one day with episodes of vomiting and constipation.

### Adverse events related to dC/dT

Diarrhea was experienced by patients 1, 8, 14, 20, 21, 22, 23, and 24. Patient 1 had mild diarrhea in the first weeks of treatment, which spontaneously resolved. Patient 22 had intermittent diarrhea at the target dose of 400 mg/kg/day. Patients 20, 21, 23, 24 had diarrhea at the target dose of 400 mg/kg/day which improved when the dose was decreased to 300 mg/kg/day. Patient 8 had a 4-day bout of diarrhea attributed to a viral infection. Patient 14 had 1 day of diarrhea in the context of starting nasogastric tube feeds.

Unintentional weight gain was reported by three adult patients. Patient 20 weighed 88.5 kg and 92.1 kg at baseline and 6 months, respectively. Patient 21 weighed 81.6 kg and 92.0 kg at baseline and 6 months, respectively. Patient 22 weighed 53.5 kg and 68.5 kg at baseline and 6 months, respectively.

Blood and urine testing did not reveal any concerning changes in end organ function. Urine purines and pyrimidines showed elevated thymine and uracil, along with elevations in their metabolites, dihydrothymine, dihydrouracil, β-ureidoisobutyric acid, β-ureidopropionic acid, and 5-hydroxymethyluracil, reflecting treatment with dC/dT [8].

### Relationship to age of symptom onset

Overall, there was no clear pattern of effectiveness being related to age of symptom onset, though analysis was of limited utility as the vast majority of patients had early symptom onset and there were few patients in the other two age of onset groups (details in supplementary data). For NMDS I-III, scores at last assessment were improved from baseline in 16/19 with symptom onset < age 12 years, 4/4 with onset 12–30 years, and 1/2 with onset >30 years. Serum GDF-15 at last assessment was decreased from baseline (when this could be evaluated) in 8/14 with symptom onset <12 years, 3/4 with onset 12–30 years, and 0/2 with onset >30 years. Normalized quality of life score (when data were available) was improved in 10/18 with symptom onset <12 years, 3/4 with onset 12–30 years, and 1/1 with onset >30 years.

## Discussion

We report long-term data from an open-label trial of dC/dT in pediatric and adult patients with POLG-related disorders. The data, derived from 530 months of cumulative exposure, suggest that dC/dT is safe in this patient population. No serious adverse effects were attributed to dC/dT. Diarrhea, the primary adverse event attributed to dC/dT, was either transient or manageable through dose reduction. Of the two primary outcomes, NMDS scores (sections I-III) decreased (improved) compared to baseline at all timepoints up to 24 months, suggesting therapeutic efficacy. The second primary outcome, serum GDF-15, was also decreased (improved) from baseline at multiple timepoints, providing additional support for efficacy from a more objective measure.

Compared to available natural history data, patients in our cohort with early-onset disease, defined previously as onset of symptoms before 12 years [15], appear to have improved survival. Hikmat et al. (2017) and Kristenson et al. (2024) reported a median time from symptom-onset to death of 4.9 months and 8 months, respectively, among patients with early-onset disease [16,17]. Based on these data, we would have expected well over half of the patients with early-onset disease in our cohort to die during the first 12 months of the trial. However, only 2/19 (11%) of patients with early-onset disease died during the first year in the trial, and only 4/19 (21%) died during the observation period, overall. Of course, comparisons to natural history data must be made with caution given the differences between cohorts with respect to care plans, era effects, and ascertainment. We also note that assessing safety is complicated in any study in which the population has severe health issues at baseline as it can be challenging to identify adverse events related to an intervention.

Five of the six adult patients in our cohort had diarrhea attributed to dC/dT. Four of these patients could not tolerate the maximum dose of 400 mg/kg/day due to this side effect. Diarrhea has been reported in eight patients with TK2 deficiency being treated with dC/dT; two of these patients, aged 28 and 32 years, required dose reduction [5]. The other four adults in their trial (aged 30–61 y) were able to tolerate 400 mg/kg/day, although a subsequent study by Hernandez-Voth et al. (2020) reported that one of these adult patients was taking a decreased dose for an unspecified reason [4]. Hernandez-Voth et al. also reported an additional patient taking a lower dose, again without specified reason. The presence of significant diarrhea among adults is possibly related to the higher doses that they receive due to weight-based dosing. In order to minimize the likelihood of this side effect in adults, it may be necessary to either use flat dosing (e.g., 8000 mg tid regardless of weight) or dose based on body surface area rather than weight.

From a mechanistic perspective, it is initially surprising that supplementation with dC and dT alone would have therapeutic benefit for POLG-related disorders. While dC and dT are the only two substrates for TK2, all four deoxynucleosides might be expected to be necessary to effectively replenish mitochondrial DNA depleted due to POLG dysfunction; however, recent *in vitro* studies have shown that supplementation with three or fewer deoxynucleosides is sufficient to increase mtDNA levels in POLG-deficient fibroblasts [18]. We only used pyrimidine deoxynucleosides in this study because the purine deoxynucleosides, deoxyguanosine and deoxyadenosine, have very low bioavailability [19,20]. However, it is possible that dC and dT act through alternate mechanisms. We recently published data showing that telomeres are shorter in people with mitochondrial DNA depletion disorders [21], and independent work has demonstrated that telomere length is highly dependent on the levels of circulating nucleotides [22]. In fact, supplementation with dT alone was sufficient to drive telomere lengthening in an *in vitro* model [23], thus telomere stabilization or elongation is one possible additional pathway through which dC/dT supplementation could elicit benefit.

Our study has several limitations. Firstly, this is an open label trial which is susceptible to potential bias, particularly from the lack of blinding. While the NMDS sections I-III scores provide an objective clinical measure, factors outside of the trial such as concurrent medical treatments can influence the scores. Patients were allowed to start, stop, or adjust other treatments during the trial, and this could influence our results. Although we sought to provide additional objective biochemical measures using serum GDF-15 levels, this biomarker has inherent limitations. First, its reported sensitivity for mitochondrial disorders is only 0.83 (10/24 patients in our study had normal baseline GDF-15 levels) [24]. Additionally, the commercial laboratory used has an upper limit of GDF-15 quantification that was exceeded by multiple samples. This prevented accurate measurement of treatment response among patients who had baseline levels above the threshold of 6000 pg/ml (Patients 8, 13, 14, and 18). Patient 8 had GDF-15 levels above this threshold throughout the trial, therefore it was not possible to determine whether there was an increase or decrease. Finally, due to circumstances outside of the control of the research team (e.g., patients’ inability to come for an in-person visit, delays in sample shipping), there are some missing data points for GDF-15 levels and other blood and urine investigations. While these issues reflect the reality of a trial of this kind, the data limitations further impact the reliability of the results, particularly given the already small sample size.

In conclusion, we present long-term data supporting the safety and efficacy of dC/dT in children and adults with POLG-related disorders. In patients with these devastating diseases, it appears that pyrimidine deoxynucleoside supplementation has the potential to change the natural history and improve survival. However, a randomized controlled trial would be the ideal approach to more clearly evaluate the therapeutic value of dC/dT in POLG-related disorders. Further research is necessary to clarify the mechanism or mechanisms by which dC/dT elicits effect, so that the intervention can be refined and optimized.

## Author contributions

SB, DB, and KAM contributed to the conception and design of the study. ACTC, CD, SB, HP, TG, PJW, RE, DB, and KAM contributed to acquisition and analysis of data. ACTC and KAM contributed to drafting a significant portion of the manuscript and figures.

## Declaration of competing interest

None.
