# Supplementary material for: Long-term safety and efficacy of deoxycytidine/deoxythymidine in treatment of POLG-related disorders
Source: Neurotherapeutics. 2026 Jun 15;23(4):e00945. doi: 10.1016/j.neurot.2026.e00945 (PMC13285371; doi:10.1016/j.neurot.2026.e00945)
Supplement: Multimedia component 2 [file mmc2.docx]

Table 1 – Patient demographics and clinical information

| **Patient #** | **Age at treatment initiation (y)** | **Sex** | ***POLG* variant(s) (NM_002693.2)** | **Clinical phenotype** | **Concurrent medications at treatment initiation** | **Nationality (Ethnic background)** |
| --- | --- | --- | --- | --- | --- | --- |
| 1 | 8.1 | M | c.2554C>T, p.(Arg852Cys);  c.1399G>A, p.(Ala467Thr) | Alpers-Huttenlocher syndrome:  Seizures (including epilepsia partialis continua), developmental impairment, ataxia, movement disorder, autism spectrum disorder. | Topiramate, carbamazepine, levetiracetam, clobazam, memantine, carnitine, leucovorin, creatine, alpha lipoic acid, liposomal ubiquinol, melatonin, vitamin D, B100 complex | Canadian (Caucasian) |
| 2 | 2.4 | M | c.1399G>A, p.(Ala467Thr); c.2740A>C, p.(Thr914Pro) | Alpers-Huttenlocher syndrome:  Seizures, stroke-like events, developmental regression. | Levetiracetam, clobazam, perampanel, leucovorin, carnitine, ubiquinol, creatine, methylated B12, multivitamin, budesonide, albuterol | American (Caucasian) |
| 3 | 8.4 | F | c.1399G>A, p.(Ala467Thr); c.1874C>T, p.(Pro625Leu) | No defined syndrome: Seizure onset at age 6 y, mild developmental impairment, liver failure requiring transplant (after treatment with valproic acid). | Phenobarbital, levetiracetam, sertraline, leucovorin, levocarnitine, sirolimus, aspirin, ursodiol, arginine, coenzyme Q10, multivitamin | American (Caucasian) |
| 4 | 6.6 | F | c.2740A>C, p.(Thr914Pro); c.1399G>A, p.(Ala467Thr) | Alpers-Huttenlocher syndrome:  Seizures, developmental regression. | Midazolam infusion, phenobarbital, topiramate, lacosamide, levetiracetam, coenzyme Q10, riboflavin, thiamine, pyridoxine, leucovorin, alpha-lipoic acid, carnitine | Canadian (Caucasian) |
| 5 | 3.3 | F | c.911T>G, p.(Leu304Arg) (homozygous) | No defined syndrome: Photophobia, constipation, mild language delay (* patient was only mildly affected at time of enrolment; diagnosed because her older brother has POLG-related disorder and is severely affected). | None | Canadian (Pakistani) |
| 6 | 17.9 | F | c.2243G>C, p.(Trp748Ser); c.1399G>A,  p.(Ala467Thr) | Ataxia neuropathy spectrum:  Depression, motor regression, fatigability, gait instability, seizures, ptosis. | Topiramate, lamotrigine, levetiracetam, phenytoin, memantine, clobazam, sertraline, omeprazole, coenzyme Q10, folic acid, B complex, riboflavin, other vitamins | Brazilian (Brazilian) |
| 7 | 11.6 | F | c1399G>A, p.(Ala467Thr) (homozygous) | Alpers-Huttenlocher syndrome:  Seizures, stroke-like episodes, developmental regression. | Levetiracetam, lamotrigine, lacosamide, brivaracetam, clobazam, gabapentin, clonidine, baclofen, famotidine, spironolactone, levocarnitine, melatonin, ferrous sulfate, lorazepam, diazepam, phenobarbital | American (Caucasian) |
| 8 | 10.3 | M | c.911T>G, p.(Leu304Arg) (homozygous) | Ataxia neuropathy spectrum: Ptosis, progressive external ophthalmoplegia, progressive muscle weakness, ataxia, areflexia | None | American (Pakistani) |
| 9 | 2.5 | M | c.911T>G, p.(Leu304Arg) (homozygous) | Alpers-Huttenlocher syndrome: Seizures, developmental regression. | Clobazam, lacosamide, phenobarbital, brivaracetam, topiramate, coenzyme Q10, riboflavin, carnitine | Indian (Indian) |
| 10 | 2.5 | M | c.2243G>C, p.Trp748Ser; c.2440_2442del, p.Val814del | Alpers-Huttenlocher syndrome: Seizures, developmental regression. Significantly worsened after inadvertent treatment with valproic acid. | Cannabidiol, brivaracetam, lacosamide, clonazepam, lorazepam, gabapentin, leucovorin, ubiquinone, omeprazole, fluticasone, carnitine, vitamin C, melatonin, vitamin D, ferrous sulfate | Canadian (Caucasian) |
| 11 | 1.0 | M | c.1947C>A, p.(Tyr649*); c.2243G>C, p.(Trp748Ser) | Alpers-Huttenlocher syndrome: Seizures, developmental regression. | Clobazam, clonazepam, levetiracetam, oxcarbazepine, phenobarbital, coenzyme Q10, levocarnitine, leucovorin, magnesium | American (Caucasian/African American) |
| 12 | 6.2 | M | c.752C>T, p.(Thr25Ile); c.2858G>A, p.(Arg953His); c.1760C>T, p.(Pro587Leu) (variant of uncertain significance) | Ataxia neuropathy spectrum:  Motor regression, muscle weakness, areflexia, ptosis. | Coenzyme Q10, mitochondrial, “Neuroneeds” mitochondrial cocktail, vitamin B12, vitamin B complex | American (Dominican) |
| 13 | 1.3 | M | c.2542G>A, p.(Gly848Ser); c.1399G>A, p.(Ala467Thr) | Alpers-Huttenlocher syndrome: Stroke-like episodes, seizures, developmental regression, visual impairment, transaminitis. | Levetiracetam, oxcarbazepine, clobazam, gabapentin, omeprazole, melatonin, intravenous immunoglobulin | American (Caucasian) |
| 14 | 14.1 | M | c.911T>G, p.(Leu304Arg) (homozygous) | Ptosis, muscle weakness, dysphagia, dysarthria, failure to thrive. | Coenzyme Q10, multivitamin | Indian (Indian) |
| 15 | 3.1 | M | c.2419C>T, p.(Arg807Cys); c.830A>T, p.(His277Leu) | Mild speech and gross/fine motor delay. | None | American (Caucasian) |
| 16 | 2.0 | M | c.3286C>T, p.(Arg1096Cys) (homozygous) | Alpers-Huttenlocher syndrome: Seizures, developmental regression. | Lacosamide, clobazam, levetiracetam, phenobarbital, perampanel, carnitine, coenzyme Q10, thiamine, biotin, arginine, glycopyrrolate, vitamin D, esomeprazole, iron, melatonin, salbutamol, budesonide, ipratropium | Emirati (Arab) |
| 17 | 3.5 | F | c.3286C>T, p.(Arg1096Cys) (homozygous) | Mild-to-moderate ataxia. | Coenzyme Q10, carnitine | Emirati (Arab) |
| 18 | 2.7 | F | c.1399G>A, p.(Ala467Thr); c.2217_2230dup, p.(Ile744Thrfs*59) | Alpers-Huttenlocher syndrome: Seizures, developmental regression, liver failure. | Phenobarbital, lacosamide, clonazepam, lansoprazole, lactulose, rifaximin, ursodiol, vitamin K1, acetylcysteine, furosemide, potassium chloride, medium chain triglycerides, coenzyme q10, thiamine, alpha-lipoic acid, levocarnitine, leucovorin, cyanocobalamin, multivitamin, intravenous immunoglobulin | American (Caucasian) |
| 19 | 21.3 | M | c.752C>T, p.(Thr251Ile); c.1760C>T, p.(Pro587Leu); c.1155C>T, p.(Arg386Cys) | Seizures, developmental regression. | Cannabidiol, levothyroxine, coenzyme Q10, lysine, leucovorin, cetirizine, vitamin E, uridine, mesalamine. | American (Jordanian) |
| 20 | 47.9 | M | c.1399G>A, p.(Ala467Thr); c.2243G>C, p.(Trp748Ser) | Ptosis, chronic progressive external ophthalmoplegia, stroke-like episodes, peripheral neuropathy, ataxia, cognitive deficits, | Aripiprazole, atorvastatin. | Canadian (Caucasian) |
| 21 | 25.3 | F | c.1399G>A, p.(Ala467Thr) (homozygous) | Seizures, stroke-like episodes, ataxia. | Cenobamate, clobazam, sertraline, coenzyme Q10, leucovorin, levocarnitine, L-arginine, norethindrone, vitamin C, vitamin D, vitamin B12, Zinc, magnesium. | American (Caucasian) |
| 22 | 19.7 | F | c.1399G>A, p.(Ala467Thr) (homozygous) | Seizures, ataxia, tremor. | Cenobamate, levetiracetam, clobazam, sertraline, coenzyme Q10, leucovorin, levocarnitine, L-arginine, medproxyprogesterone, vitamin C, vitamin D, vitamin B12, Zinc, magnesium. | American (Caucasian) |
| 23 | 18.4 | F | c.1399G>A, p.(Ala467Thr) (homozygous) | Seizures, stroke-like episodes, ptosis, chronic progressive external ophthalmoplegia, ataxia. | Levetiracetam, lacosamide, lamotrigine, sertraline, vitamin D, vitamin B6, melatonin. | Canadian (Caucasian) |
| 24 | 28.7 | M | c.1399G>A, p.(Ala467Thr) (homozygous) | Ataxia neuropathy spectrum:  Seizures, peripheral neuropathy, ptosis, ataxia, chronic progressive external ophthalmoplegia. | Lamotrigine, topiramate, coenzyme Q10, leucovorin, levocarnitine, thiamine, lansoprazole, riboflavin, aquasol. | Canadian (Caucasian) |
| 25 | 39.6 | F | c.1399G>A, p.(Ala467Thr) (homozygous) | Dystonia, ataxia, peripheral neuropathy, ptosis, chronic progressive external ophthalmoplegia. | Lorazepam, zaleplon, glycopyrrolate, sertraline, clonazepam. | American (Caucasian) |
